# Emotion regulation among bariatric seeking patients with obesity and food addiction: a cross-sectional, unmatched nested case–control comparison

**DOI:** 10.1186/s40337-025-01424-6

**Published:** 2025-12-15

**Authors:** Lou Madieta, Amandine Scocard, Marine Rozet, Arnaud De Luca, François Kazour, Servane Barrault, Nicolas Ballon, Bénédicte Gohier, Paul Brunault

**Affiliations:** 1 Service de Psychiatrie et d’Addictologie, CHU d’Angers, 4 rue Larrey, Angers, 49100 France; 2 Service d’Addictologie Universitaire, CHRU de Tours, Équipe de Liaison et de Soins en Addictologie, Tours, France; 3 CHRU de Tours, Centre Spécialisé de l’Obésité, Tours, France; 4 Université de Tours, Inserm U1069, Tours, France; 5 Université d’Angers, Nantes Université, [CHU Angers], LPPL, SFR CONFLUENCES, F-49000, Angers,, France; 6 CHRU de Tours, Centre de Soins d’Accompagnement et de Prévention en Addictologie d’Indre-et-Loire (CSAPA-37), Tours, France; 7 Qualipsy EE 1901, Université de Tours, Tours, France; 8 Imaging Brain & Neuropsychiatry iBraiN U1253, Université de Tours, INSERM, Tours, 37032 France

**Keywords:** Food addiction, Eating disorders, Obesity, Emotional regulation, Cognitive behavioral therapy

## Abstract

**Background:**

Emotion dysregulation, alexithymia and attentional biases toward food or emotional stimuli have been reported in patients with obesity and food addiction (FA), but the relative contribution of obesity or FA to these characteristics remains unclear. Our objectives were to compare patients with obesity and FA, patients with obesity without FA and patients without obesity regarding cognitive emotion regulation strategies, alexithymia, emotion regulation difficulties, and attentional biases.

**Methods:**

We included 37 bariatric seeking patients (18 FA, 19 without FA) and 37 controls in a cross-sectional, unmatched nested case–control design. We assessed food addiction (YFAS 2.0), emotional regulation strategies (CERQ), emotion regulation difficulties (DERS), alexithymia (TAS-20), and attentional biases (Stroop and Emotional Stroop tasks).

**Results:**

Among patients with obesity, those with FA differed from non-FA only in terms of cognitive emotion regulation strategies: less refocus on planning (*p* = .04), more catastrophizing (*p* = .02), and more positive refocusing (*p* < .001). Patients with obesity (with or without FA) presented higher scores regarding emotion regulation strategies (*p* < .05), alexithymia (*p* < .001) and emotion regulation difficulties (*p* < .001). Neither obesity nor FA were associated with attentional bias toward food or negative emotional stimuli and cognitive inhibition.

**Conclusions:**

Among patients with obesity, having a FA was related to cognitive avoidance toward negative events, but not to change in the saliency of emotional or food stimuli. Alexithymia was more related to obesity than to FA.

**Graphical abstract:**

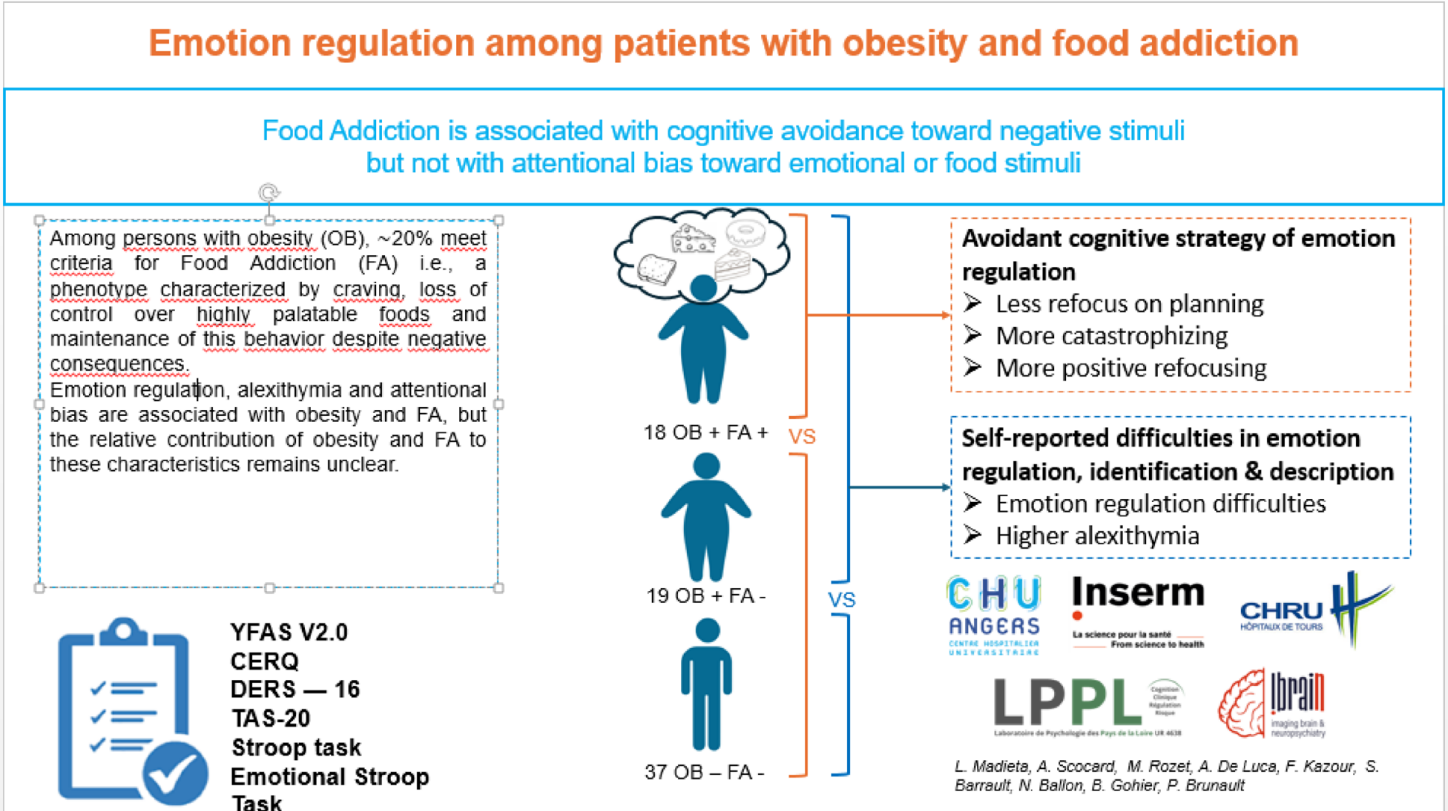

**Supplementary Information:**

The online version contains supplementary material available at 10.1186/s40337-025-01424-6.

## Abstract (plain language summary)

This study looked at how people with obesity, with or without food addiction, manage their emotions and how they pay attention to emotional or food-related cues. We wanted to find out whether food addiction or obesity played a bigger role in how people experience emotional difficulties and how they cope. We compared three small groups: people with obesity and food addiction, people with obesity without food addiction, and people without obesity. We found that people with obesity—regardless of food addiction—had more trouble understanding and managing their emotions than those without obesity. However, those with both obesity and food addiction were more likely to think unhelpful thoughts, such as expecting the worst or avoiding planning how to cope with challenges. Interestingly, the study found that neither food addiction nor obesity seemed to change how people paid attention to emotional or food-related images. The findings suggest that emotional challenges may be more strongly linked to obesity itself, rather than food addiction. These insights may help guide more targeted support for people with obesity, especially when it comes to emotional health and coping strategies.

**Supplementary Information:**

The online version contains supplementary material available at 10.1186/s40337-025-01424-6.

## Introduction

Obesity is a global pandemic concerning 13.1% of worldwide adults, a prevalence doubled since the 1990 [1]. Obesity is associated with an elevated risk of mortality, lower quality of life and higher prevalence for co-occurrent psychiatric disorders and general medical disorders [2–4]. Tackling obesity and its associated factors is one of the prioritized target of the World Health Organization [5]. To this end, identifying subgroups of vulnerable patients among people with obesity helps design better tailored treatments.

Obesity is associated with a wide range of biopsychosocial factors, including genetic aspects, limited physical activity, excessive calorie intake that may be due to high in fat and or sugar foods, and disordered eating [6]. In line with this later hypothesis, food addiction (FA) has been proposed as a risk factors for obesity in a subgroup of patients [7–9]. Approximately 25% of people with obesity or overweight suffer from FA [10]. FA is defined by the use of highly palatable food in a pattern similar to substance use in substance use disorder [11]. This includes the presence of tolerance, withdrawal symptoms, larger amounts consumed than intended, persistent desire or unsuccessful attempts to cut down, much time spent using or recovering from food usage, continual use despite knowledge of adverse consequences or activities given up due to use of food.

In FA, part of the food intake may be emotionally driven [12], as it was already demonstrated in subgroups of patients with classical eating disorders [13] or substance use disorder [14]. As proposed in the self-medication theory, eating these foods may be a coping strategy [15, 16]. As in substance abuse, difficulties in emotion regulation may increase the risk of regular high palatable food intake onset and maintenance leading to eating disorders including FA [17–19]. According to the model of emotional regulation processes [20], emotion regulation can occur at behavioral (situation modification), attentional (attentional deployment) and cognitive levels (cognitive change ) levels. Furthermore, when evaluating emotional regulation via subjective measures such as self-administered questionnaires, it is necessary to account for the capacity to identify and express emotion (i.e., alexithymia). Such a multilevel assessment of emotion regulation processes in obesity and FA may provide valuable insight to better design tailor-based treatment for these patients.

On one hand, previous works showed that at the cognitive level, obesity was associated with more frequent use of cognitive emotion regulation strategy such as refocus on planning and catastrophizing [21]. Individuals with obesity also showed more intense alexithymia and difficulty in emotional awareness and emotion regulation strategies, namely, reduced cognitive reappraisal and acceptance, and greater suppression of emotion expression [22]. At the attentional level, while the literature is heterogenous, a majority of published studies showed an attentional bias toward food cues [23]. These works did not account for the presence of FA in studied samples.

On the other hand, previous work conducted among patients with FA showed that it was associated with more difficulty in emotion regulation in aspects of lack of awareness of emotional responses, non-acceptance of emotional responses, lack of clarity of emotional responses, limited access to emotion regulation strategies, impulse control difficulties, difficulties engaging in goal-directed behaviors [9]. FA was also associated with impulsivity both in terms of cognitive aspects and non-planning impulsivity [24], but also with alexithymia [25]. Attentional bias toward food-related cues in FA may be altered by emotional stimuli as it’s been shown that a sad mood increases attentional bias toward unhealthy highly palatable foods [26]. These works did not account for the presence of obesity in studied samples.

In sum, obesity and FA are both associated with emotion dysregulation and alexithymia, but the relative contribution of these mechanisms to obesity and FA remains unclear. In addition, investigating attentional biases toward food or negative emotional stimuli may provide additional knowledge of the mechanisms underlying obesity or FA, but studies in this field are lacking.

The main objective of our study was to compare patients with obesity and FA versus patients with obesity but no FA in terms of cognitive emotion regulation strategies. We hypothesized that among patients with obesity, having a FA may be associated with more non-adaptive and less adaptive cognitive emotion regulation strategies. Our secondary objectives were: (1) to compare patients with obesity and FA versus patients with obesity but no FA in terms of other emotion regulation-related variables (i.e., difficulties in emotion regulation, alexithymia, attentional bias toward food or negative emotion-related stimuli, cognitive inhibition); (2) to compare these patients with obesity (with or without FA) versus patients without obesity (control group) regarding the same emotion regulation-related variables. We hypothesized that there may be a gradient of increased severity between patients without obesity, patients with obesity but no FA, and patients with obesity and FA in terms of alexithymia, difficulties in emotion regulation, cognitive inhibition and attentional bias toward food or negative stimuli.

## Materials and methods

### Participants and procedures

In this cross-sectional study, we recruited persons using a consecutive sampling from two groups: (1) a group of patients with obesity (BMI ≥ 30 kg/m^2^) consulting in the Department of Psychiatry and Addiction at the University Hospital of Tours, France that were recruited between May 2022 and November 2022 (patients referred to psychiatrists from the Specialized Obesity Center of the same hospital), and (2) an unmatched control group constituted of adults without obesity (BMI ≤ 30 kg/m²) recruited between December 2022 and July 2023 from a non-clinical population (university members and students). Patients were eligible for the study if they were at least 18 years old, were willing to participate and had a BMI ≥ 30 kg/m². In both populations, exclusion criteria were similar: participants were excluded if they presented any bias inducing disability (including motor, perceptive or cognitive impairment), if French was not their native language or if they had significant difficulties in answering the self-administered questionnaire. In the patient group, the study inclusion was systematically proposed by the psychiatrists in charge of the patient follow-up (PB or AS), and patients were assessed during a research consultation scheduled in the following month.

All groups underwent a single consultation assessment with the same physician (LM). This consultation included data collected from medical records, self-administered questionnaires as well as a neuropsychological task specifically designed for this study using the Psychopy software [27] (see Supplemental Material A for more details).

### Measures

#### Sociodemographics and medical characteristics

In both groups, we collected the following data using self-administered questionnaires or medical records: demographics (age, gender, marital and employment status), past and current medical and psychiatric disorders to assess exclusion criteria. height and weight were extracted from medical record in the patient group and self-reported for the control group.

#### Food addiction

We assessed FA diagnosis and the severity of FA using the Yale Food Addiction Scale 2.0 (YFAS 2.0) (original version [11]; French validation [28]). This self-administered questionnaire assesses addictive-like symptoms of eating high in fat and or sugar foods (especially highly processed) during the past 12 months, based on DSM-5 criteria for substance use disorder. The YFAS can be used both as a severity scale and a diagnostic scale with a dichotomous rating according to specifically defined cut-offs [29]. In this study, we assessed both the existence of a FA diagnosis as well as the number of FA symptoms experienced during the previous 12 months (score ranging from 0 to 11). In our study, both Cronbach’s α and McDonald’s ω was excellent at 0.96. In the French version validation study McDonald’s ω was 0.86 [28]).

#### Cognitive emotion regulation strategies

We assessed emotion regulation strategies using the French version of the Cognitive Emotional Regulation Questionnaire (CERQ) (original version : [30]; French validation : [31]). The CERQ is a self-report questionnaire evaluating nine distinct emotional regulation strategies in response to an adverse event [31]. A higher score reflects a more frequent use of this type of emotional strategy. These nine strategies were regrouped by the original authors as adaptive (acceptance, positive refocusing, refocus on planning, positive reappraisal, putting into perspective) or non-adaptive (self-blame, rumination, catastrophizing, blaming others). In our study, Cronbach’s α and McDonald’s ω for the Adaptive subscore was 0.91 (in the French version validation study McDonald’s ω was 0.89 [31]), for the non-adaptive subscore Cronbach’s α was 0.85 and McDonald’s ω was 0.86 (in the French version validation study McDonald’s ω was 0.82 [31]).

#### Emotion regulation difficulties

We assessed emotion regulation difficulties using the French version of Difficulty in Emotion Regulation Scale (DERS-16) (original long version : [32]; original short version : [33]; French validation: [34]). In this study, we used total DERS score as indicator of emotion regulation difficulties, a higher score indicating more difficulty in regulating emotions and McDonald’s ω were excellent at respectively 0.94 and 0.95. In the French version validation study, Cronbach alpha was 0.94 [34]).

#### Alexithymia

We assessed alexithymia using the Toronto alexithymia scale (TAS-20) (Original version : [35] French validation : [36]). The TAS-20 provides a total score and three sub-scores: difficulty identifying emotions, difficulty describing emotions and operational thinking. A higher total score on the TAS-20 indicates higher alexithymia. In the French version validation study, Cronbach α was 0.79 [36]. In our study, Cronbach α and McDonald’s ω f or the total TAS-20 score were good at respectively 0.85 and 0.86. More specifically, Cronbach α and McDonald’s ω were respectively at 0.76 and 0.77 for the subscale difficulty identifying emotions, 0.78 and 0.79 for difficulty describing emotions finally 0.53 and 0.49 for operational thinking.

#### Cognitive inhibition, and attentional bias toward food and negative emotional stimuli

Participants cognitive inhibition and attentional biases were assessed using a neuropsychological assessment that included a Stroop task (Color-Word Interference Test) [37] and an emotional Stroop task [38, 39] specifically designed for this study with evaluation of attentional bias toward negative emotional stimuli and food stimuli (the detailed procedure is available in the Supplemental Material A). The Stroop task enables the calculation of a difference in mean response time in congruent conditions (words written in the corresponding color) and non-congruent conditions (words written in a non-corresponding color) which reflect the ability to suppress contradictory information (e.g., the written word meaning). The emotional Stroop task enables the calculation of a difference in mean response time in emotional condition (food-related stimuli and emotion-related stimuli) and in mean response time in neutral condition, which reflect the ability to suppress emotional related information.

### Statistical analysis

Statistical analyses were performed using the JASP 0.17.3 software. All analyses were two-tailed; p-values < 0.05 were considered statistically significant. Missing data were excluded from the analysis. Descriptive statistics included percentages for nominal variables and means and standard deviations for continuous variables.

To test our main hypothesis, we used Kruskal-Wallis tests to compare the three groups (patients with FA and obesity, patients with obesity and without FA, control group). Group was the factor; outcomes were dependent variables, namely sociodemographic characteristics, CERQ sub-score, DERS total score, TAS total score and sub-scores, Stroop effect (cognitive inhibition measured by the difference in mean response time between congruent and non-congruent condition at the Stroop task), food valence (attentional bias toward food measured by the difference in mean response time between food related stimuli task and neutral stimuli at the emotional Stroop task) and emotional valence (attentional bias toward emotional stimuli measured by difference in mean response time between negative emotional stimuli and neutral stimuli at the emotional Stroop task). Effect size was expressed using partial eta squared (ηp²).

Due to the exploratory nature of our work, we could not estimate a needed sample size due to the and targeted a minimum of 30 patients per groups.

To test our secondary hypotheses, we used Kruskal-Wallis tests when the independent variables were continuous, we expressed effect size as partial eta squared (ηp²). we used chi-squared tests when the independent variables were nominal. We expressed effect size as Cramer’s V To assess the significant difference of Stroop effect, Food valence and Emotional valence tasks compared to a neutral reaction (mean reaction time would thus be equal to 0), we used a Wilcoxon signed-rank with the null hypothesis being “there was no difference between the two respective conditions. Due to the limited sample size and the associated lack of power, we could not check for interaction analysis for socio-demographic characteristics such as gender.

### Ethics

This study has been approved by the institutional review board of the University Hospital of Tours (N°:2022_028) for the clinical population and by the institutional review board of the University and Tours and Poitiers (Comité d’Éthique pour les Recherches sur la Personne Tours- Poitiers) (CER-TP 2022-09-01), prior to the beginning of the study. All procedures were performed in accordance with the ethical standards of the national and institutional research committee as well as with the 1964 Helsinki declaration and its later amendments or comparable ethical standards. All participants provided informed consent after the procedure had been fully explained and prior to their inclusion in the study. All collected data were in line with the French recommendation regarding use of personal data, with the approval of the French “Commission Nationale de l’Informatique et des Libertés”.

## Results

### Study flow chart

**Fig. 1:**
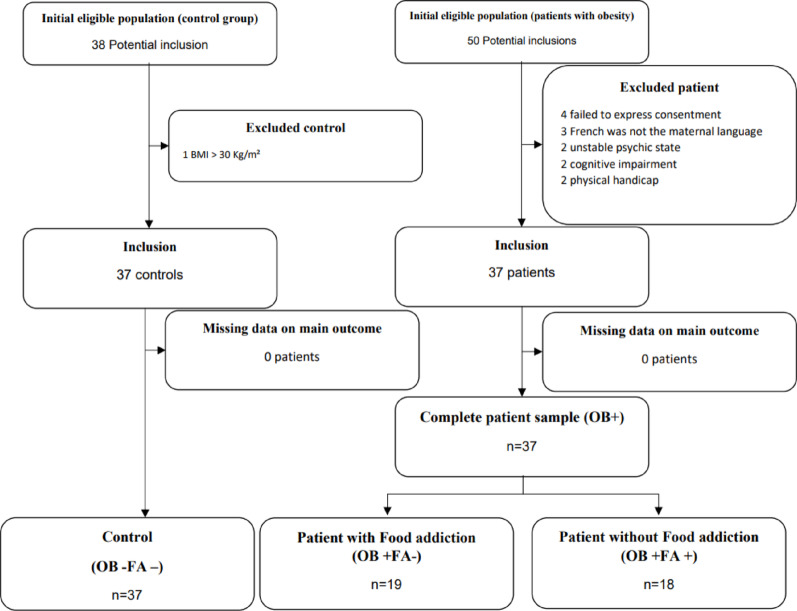
Study flow chart

Figure 1 presents the study flow chart. Among the eligible persons in the patient group *n* = 50), four patients refused to participate, nine presented an exclusion criterion. Among the eligible persons in the control group, one control presented an exclusion criterion. This led to a total sample of 74 included participants (i.e., 37 patients and 37 controls).

### Descriptive statistics

#### Sociodemographic and medical characteristics

The sociodemographic and medical characteristics of our sample are presented in Table 1. Mean age was 40.0 ± 14.1; Mean BMI was 32.9 ± 13.9 kg/m^2^. 74.3% % (55) of participants were female.

(Table 1)

**Table 1:** Descriptive statistics of our study sample regarding sociodemographic and medical characteristics, and comparison between the three groups in univariate analyses

	Complete sample (n=74)	OB+ FA+ Group (n=18)	OB+ FA- Group (N= 19)	OB- FA- Group (n=37)	P (effect size)	Z statistics for Pair-wise comparaison (p-value)		
						OB+ FA+ vs. OB+ FA-	OB+ FA- vs. OB- FA-	OB+ FA+ vs. OB- FA-
Age (Year)	40.0±14.1	41.1±16.3	48.4±12.0	35.1±12.0	<.001(0.15)	1.6 (.11)	3.3 (<.001)	1.4 (.16)
BMI (Kg/m²)	32.9±13.9	45.6±7.8	39.9±7.4	23.1±3.0	< 0.001(0.52)	− 0.6 (.57)	5.8 (<.001)	6.3 (<.001)
Gender (Female)	74.3% (55)	100 % (18)	63.2% (12)	67.6% (25)	0.02 (0.34)			
Familial status (relationship)	64.9% (48)	50 % (9)	69.6 % (39)	75.7% (28)	0.15-(0.21)			
Professional status (employed)	64.9% (48)	44.4% (8)	42.1% (8)	86.5% (32)	< 0.001(0,45)			
Number of FA symptoms (YFAS 2.0)	2.8±3.3	7.3±2.4	2.5±2.5	0.8±1.4	< 0.001(0.65)	− 3.6 (<.001)	2.3 (.02)	6.5 (<.001)

Continuous variables were compared between the three groups using Kruskal Wallis test with ηp² for effect size; nominal variables were compared using chi-squared tests with Cramer’s V as effect size. YFAS 2.0: Yale Food Addiction Scale 2.0

#### Cognitive emotion regulation strategies, emotion regulation difficulties and alexithymia

Emotion regulation specificities and alexithymia are presented in Table 2. For the patient group, concerning cognitive emotion regulation strategy use, mean use of adaptive emotional regulation strategy was 61.0 ± 19.6 for the OB + FA-; 52.72 ± 11.0 for the OB + FA+. Mean use of non-adaptive emotional regulation strategy was 33.3 ± 9.3 for the OB + FA- and 41.2 ± 8.3 for the OB + FA+. Concerning emotion regulation difficulties, mean total DERS score was 25.8 ± 15.4 for the OB + FA- and 30.1 ± 13.9 for the OB + FA. Concerning alexithymia, mean alexithymia intensity was 50.9 ± 11.4 for the OB + FA- and 55.9 ± 12.0 for the OB + FA+.

For the control group, concerning cognitive emotion regulation strategy use, mean use of adaptive emotional regulation strategy was 63.2 ± 14.0. Mean use of non-adaptive emotional regulation strategy was 36.0 ± 10.2. Concerning emotion regulation difficulties, mean total DERS score was 15.5 ± 13.6. Mean alexithymia score was 44.1 ± 12.8.

**Table 2:** Descriptive statistics of our study sample regarding cognitive emotion regulation strategies, emotion regulation difficulties and alexithymia, and comparison between the three groups in univariate analyses

	Complete sample (n=74)	OB+ FA+Group(n=18)	OB+ FA- group(n= 19)	OB- FA-group(n=37)	p(effet size)	Z statistics for pair-wise comparaison (p-value)		
						OB+ FA+ vs. OB+ FA-	OB+ FA- vs. OB- FA-	OB+ FA+ vs. OB- FA-
Adaptive emotional regulation strategy total score (CERQ)	60.1±15.4	52.72±11.0	61.0±19.6	63.2±14.0	0.03 (0.08)	1.9 (.06)	− 0.4 (.68)	− 2.6 (<.001)
Acceptance (CERQ1)	13.4±3.8	12.4±3.9	13.6±5.0	13.8±3.0	0.55 (0.02)			
Positive refocusing (CERQ2)	10.8±4.2	13.8±3.6	9.3±4.3	10.0±3.9	0.25 (0.04)			
Refocus on planning (CERQ3)	11.3±4.6	7.2±2.0	10.3±5.0	13.9±3.6	< .001(0.37)	2.1 (.04)	− 2.9(.004)	− 5.2 (<.001)
Positive reappraisal (CERQ4)	12.4±4.0	11.0±4.0	13.0±4.6	12.8±3.7	<.001(0.17)	− 3.1 (<.001)	− 0.6 (.57)	3.0 (<.001)
Putting into perspective (CERQ5)	12.0±4.0	10.6±3.8	11.7±4.7	12.8±3.6	0.17 (0.05)			
Non- adaptive emotional regulation strategy total score (CERQ)	36.6±9.9	41.2±8.3	33.3±9.3	36.0±10.2	0.05 (0.08)	− 2.3 (.02)	− 0.8 (0.4)	1.9 (0.06)
Rumination (CERQ6)	11 .0±4.0	10.3±2.9	9.7±4.2	11.9±4.2	0.06 (0.06)			
Self-blame (CERQ7)	8.9±3.6	8.0±3.53	7.2±3.4	10.2±3.2	<.001(0.14)	− 0.8 (0.4)	− 3.2(.001)	− 2.2 (0.03)
Blaming others (CERQ8)	9.6±4.5	11.6±4.2	12.5±4.8	7.2±3.1	<.001(0.29)	0.3 (0.8)	4.0 (<.001)	3.6 (<.001)
Catastrophizing (CERQ9)	7.4±3.1	9.1±2.9	7.1±3.5	6.8±2.8	0.01 (0.09)	− 2.3 (0.02)	0.2 (0.8)	2.8(.005)
Difficulty in emotion regulation (DERS total score)	21.7±15.4	30.1±13.9	25.8±15.4	15.5±13.6	<.001(0.17)	− 0.8 (0.44)	2.6 (<.01)	3.4 (< .001)
Alexithymia total score (TAS-20)	48.7±13.1	55.9±12.0	50.9±11.4	44.1±12.8	005(0.15)3	− 1.0 (0.29)	1.9 (0.05)	3.1 (<.001)
Difficulty identifying emotions (TAS-20)	17.4±6.0	21.0±5.0	18.7±5.2	15.0±5.8	0.0013 (0.18)	− 1.2 (0.22)	2.1 (0.03)	3.5 (<.001)
Difficulty describing emotions (TAS-20)	13.8±5.0	16.3±5.2	13.8±4.6	12.5±4.8	0.03 (0.10)	− 1.5 (0.14)	1.0 (0.31)	2.7(.007)
Operational thinking (TAS-20)	17.5±4.6	18.6±5.3	18.4±4.2	16.5±4.4	0.10 (0.05)			

Continuous variables were compared between the three groups using Kruskal Wallis test with ηp² for effect size; nominal variables were compared using chi-squared tests, effect size were expressed using Cramer’s V. CERQ=Cognitive emotion regulation questionnaire; DERS = Difficulties in Emotion Regulation Scale; TAS-20=Toronto Alexithymia Scale, 20 items

#### Attentional bias and cognitive Inhibition

Results of the neuropsychological assessments are presented in Table 3. Concerning attentional bias, mean Emotional Valence in the whole sample was − 1.2 ± 11.0 ms; it did not significatively differ from 0 (*p* = .35). Mean Food Valence was 1.3 ± 12.3 ms; it did not significatively differ from 0 (*p* = .35). Concerning cognitive inhibition, mean Stroop Effect was 10.4 ± 26.5 ms; it significatively differed from 0 (*p* < .001).

**Table 3:** Descriptive statistics of our study sample regarding attentional biases and cognitive inhibition, and comparison between the three groups in univariate analyses

	Complete sample (n=74)	OB+ FA+ group (n=18)	OB + FA – group (n= 19)	OB– FA – group (n=37)	Difference from 0 U (p-value)	Subgroup comparison p (effect size)
Emotional valence (ms)	− 1.2±11.0	1.2±14.9	− 3.5±10.0	− 1.2±9.3	1106. 00 (0.13)	0.85 (0.02)
Food valence (ms)	1.3±12.3	0.7±14.8	2.3±13.6	1.1±10.5	1390.00 (0.99)	0.89(0.002)
Stroop effect (ms)	10.4±26.5	6.9±35.2	6.8±29.1	14.0±19.8	2091.00 (<.001)	0.97 (0.02)

Significant difference from 0 was calculated using Wilcoxon signed-rank; Continuous variables were compared between the three groups using Kruskal Wallis test with ηp² for effect size

### Comparison between the three groups in univariate analyses

(Table 2)

#### Socio-demographics characteristics

Our subgroups presented significant differences in terms of age (*p* < .001; ηp^2^ = 0.15), BMI (*p* < .001; ηp²=0.52)), gender (*p* = 0,02; ηp²=0.34), professional status (*p* < .001 ηp²=0.45) and FA symptoms (*p* < .001 ηp²=0.65). Cognitive emotion regulation strategies, emotion regulation difficulties and alexithymia.

In terms of main effects, univariate analyses showed differences between adaptive emotional regulation strategy and non-adaptive emotional regulation strategy between the three groups (significant main effects with *p* = .03; ηp²=0.08 and *p* = .05; ηp²=0.08, respectively), as well as significant differences in terms of difficulties in emotion regulation (*p* < .01; ηp²=0.17) and alexithymia (*p* < .001; ηp²=0.15).

**Fig. 2:**
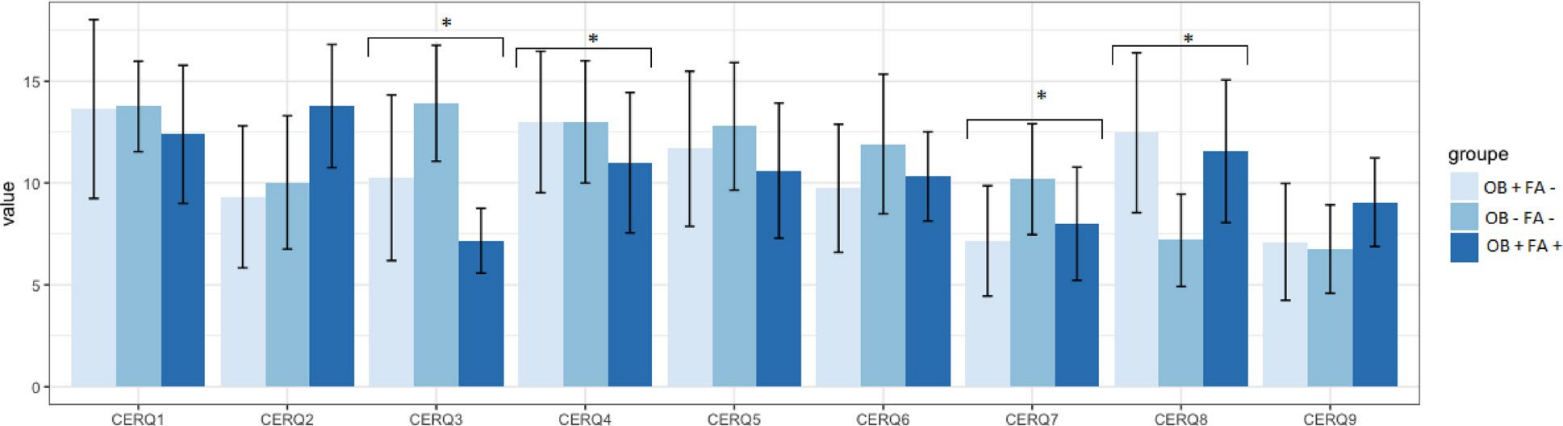
Adaptative and non-adaptative cognitive emotional regulation strategy. Acceptance (CERQ1); Positive refocusing (CERQ2); Refocus on planning (CERQ3); Positive reappraisal (CERQ4); Putting into perspective (CERQ5); Rumination (CERQ6); Self-blame (CERQ7); Blaming others (CERQ8); Catastrophizing (CERQ9)

Our primary objective are illustrated in Fig. 2 (comparison between OB + FA + and OB + FA - patients), we found that OB + FA + patients reported significantly less frequent use of refocus on planning (p=.<0.01; ηp²=0.37), and more frequently the use of catastrophizing (*p* = .01; ηp²=0.09) and positive reappraisal (*p* < .001; ηp²=0.17) than OB + FA - patients. The two groups did not differ in terms of other cognitive emotion regulation strategies. The OB + FA + group did not differ from the OB + FA – group in terms of difficulty in emotion regulation (*p* = .44) or in terms of alexithymia (*p* = .29). When considering either all adaptive or all non-adaptive emotion regulation strategies altogether, post-hoc analyses showed that the OB + FA + subgroup used more frequently non-adaptive emotional regulation strategies than the OB- FA+ (*p* = .02) but no significant differences were observed in terms of use of adaptive emotional regulation strategies (*p* = .06).

To answer our secondary objective (comparison between persons with obesity versus without obesity), we found that both patients from the OB + FA + and OB + FA – groups reported significant higher difficulties in emotion regulation, higher alexithymia and different use of some cognitive emotion regulation strategies than the normal weight group, namely lower refocus on planning, higher use of blaming others and lower self-blame. Patients from the three groups did not differ in terms of the other cognitive emotion regulation strategies, namely acceptance, positive reappraisal, putting into perspective, or rumination. When considering either all adaptive or non-adaptive emotion regulation strategies altogether, post-hoc analyses showed no significant differences in terms of non-adaptive strategies between the OB + FA + and OB – FA – subgroups (*p* = .06) or between the OB + FA – and OB – FA – subgroups (*p* = .40); there were no significant differences in terms of adaptive strategies between the OB + FA – and the OB – FA – subgroups (*p* = .68), but a lower use of adaptive strategies in the OB + FA + subgroup when compared to the OB – FA – subgroup (*p* < .001).

#### Cognitive Inhibition and attentional biases toward food or negative emotional stimuli

There were no significant differences in attentional biases between the three subgroups both in terms of attentional bias toward negative emotional stimuli (Emotional valence were respectively 1.2 ± 14.9, -3.5 ± 10.0, -1.2 ± 9.3 for the OB + FA + group, OB + FA – group and OB- FA – group; *p* = .44) and attentional bias toward food stimuli (mean Food valence were respectively 0.7 ± 14.8, 2.3 ± 13.6, 1.1 ± 10.5 for the OB + FA + group, OB + FA – group and OB- FA – group; *p* = .91). There were no significant differences in Cognitive inhibition between the sub-groups (*p* = .97).

## Discussion

Our study main result was that among patients with obesity, having a FA was associated with some specific cognitive emotional regulation strategies (CERQ scores): less refocus on planning, more catastrophizing, and more positive refocusing. Counterintuitively, patients with obesity and FA did not differ from those without FA in terms of emotion regulation difficulties (DERS score) or alexithymia (TAS-20 scores). Emotion regulation difficulties and two dimensions of alexithymia (i.e., difficulties in identifying and in describing emotions) were associated with obesity rather than with FA. Finally, we found no significant differences between our three groups of patients in terms of attentional bias toward food or negative emotional stimuli and cognitive inhibition.

In preceding work, the presence of FA has been associated with poorer executives functions [40, 41], impulsivity [42], more severe psychopathological trait [43, 44]. Less efficacy of therapeutic interventions [45, 46]. Concerning eating behavior, FA has been associated with an increase of emotional eating frequency [44]. This aspect can be explained by our founding that, among patients with obesity, having a FA was associated with two non-adaptive cognitive emotion regulation strategies: less refocus on planning and more catastrophizing. Previous study in obese sample also found similar variation of refocus on planning use and catastrophizing variation according to BMI without accounting for FA [21].

Eating highly palatable foods may be one effective (short-term) coping strategy to avoid negative thoughts or negative emotions associated with the exposition (real or anticipated) to the thoughts associated with negative events meaning that FA is associated with cognitive avoidance toward negative events. More catastrophizing and less refocus on planning may in fact be due to the same psychopathological mechanism: a cognitive avoidance of the thoughts associated with the exposition to negative events (i.e., catastrophizing refers to the terror of the experience related to negative events) as well as cognitive avoidance of the anticipation of the exposition to these events (i.e., refocus on planning refers to thinking about what steps to take in order to deal with the negative event). In both cases, the thoughts associated with the *exposition* or *anticipation of* the exposition to negative events may overtake the adaptative capacities of one’s individual and may lead to more cognitive avoidance, thus impeding the patient’s perceived self-efficacy to cope with all types of negative events.

In our study, we found that the other non-adaptive strategies (i.e., rumination, self-blame, blaming others) were not associated with FA. Studies conducted in patients with other addictive disorders found divergent associations with non-adaptive emotions (i.e., more frequent use of self-blame and blaming others) [47, 48]. While these divergent results may be explained by the differences in included population; it may be due to a difference in nature of the cognitive emotion regulation strategies assessed: self-blame and blaming others refer to the attribution style of a person toward negative events ; catastrophizing and refocus on planning refer to a direct cognitive exposition to negative affective state or experience, which the patient with FA may try to avoid.

Our results are compatible with classical models of binge eating developed from sample of patient with binge eating disorder (BED), namely some parts of the escape theory of binge eating (which postulates that some persons may develop binge eating because of a desire to escape from self-awareness; in this case, escape from self-awareness specifically related to negative events) [49] and the emotion regulation model of binge eating (which posits that patients with Binge Eating Disorder have deficits in emotion regulation processes and difficulty in regulating their negative emotions, binge eating being a way to cope with these emotions and to find relief) [50]. Catastrophizing has been shown to be the sole cognitive strategy associated with binge eating disorder [51].In line with these findings, we may assume that FA may be more strongly linked to thoughts associated with the direct affective exposition to negative events rather than to indirect affective exposition such as attribution theory.

Surprisingly, having a FA was associated with one adaptive strategy in our study: more use of positive refocusing. Which differ from results in sample of people with moderate or severe obesity [21] or substance use disorder [52]. Similar use of this strategy has also been demonstrated in patients with gambling disorder [47, 53].

Two hypotheses may be proposed: as proposed by Ruiz de Lara in gambling disorder [53], the short term efficacy of the strategies may reinforce biased beliefs about the outcomes of the addictive behavior (in the case of gambling, beliefs about gambling outcomes and controllability; in the case of food, biased beliefs about the positive outcomes of eating). Another explanation could be that in this case positive refocusing is a mean to avoid the exposition to thoughts and affect associated with negative events and these questions the adaptive nature of this cognitive strategy. When overused, positive refocusing might be another facet of lower focus on planning: cognitive avoidance toward negative events.

Another interesting result of our study was that in our sample patients with obesity presented more difficulty in emotion regulation (DERS total score) than controls regardless of the presence of FA. These results are in line with complementary work showing that in general population, people at high FA risk (without accounting for BMI) or with a higher BMI (without accounting for FA) show more difficulty in emotion regulation [21, 54]. A precedent work in pre-bariatric sample showed that people with obesity and FA presented more difficulty in emotion regulation that people with obesity without FA [12]. Emotional eating, which has been linked to Emotional regulation difficulty [55], has been shown to be a meeting point of FA and BED [56]. Altogether these results are in line with a separate association of obesity and FA to difficulty in emotion regulation with stronger relative association strength between obesity and difficulty in emotion regulation and advocate for complementary work measuring relative correlation association on the subject. As observed for emotion regulation difficulties, patients with obesity had higher alexithymia than controls, without a significative difference between patients with or without FA. The association between obesity and alexithymia is in line with the literature as patient with obesity presented more alexithymia especially more difficulty in identifying emotions and an externally oriented thinking style [22]. Li et al. work found a significant correlation between alexithymia and FA severity [25]. Altogether these works show that alexithymia may be more closely linked to obesity than to FA and as such may not be a direct therapeutic target when focusing FA. As our sample was constituted of patients with severe obesity (mean BMI was 45.6 and 39.9 in our FA and non-FA samples), future studies should be conducted in patients with less severe obesity before we may generalize our findings to the broader population of patients with obesity.

In our study, cognitive inhibition was not specifically altered in OB + FA + when measured by the Stroop task [57]. These results are in line with precedent studies exploring the association with FA and deficit in inhibition control in similar or different populations [40, 41, 57, 58]This show a difference between FA and substance use disorders in which there seems to be a causal association between inhibition control deficit and the onset of chronic substance use [17–19, 59, 60]. FA was not specifically associated with cognitive inhibition deficit in obese patients, and results from cross-sectional and longitudinal studies support the idea that inhibition deficits may be more due to obesity than FA [61]. Our work failed to demonstrate a specific attentional bias toward neither food nor negative emotion stimuli in patients with obesity and FA which. A precedent work using image cues showed a preference toward unhealthy food cues population of patient with FA without obesity [26] and Cognitive bias modification therapies applied to unhealthy eating stimuli seems to be an effective adjunct therapy in weight loss interventions [62]. which seems to advocate for a false negative in this aspect of our study .

We may assume that the deficits in emotion regulation observed in patients with obesity and FA may be more linked to explicit emotion regulation (i.e., as assessed by the CERQ) than implicit emotion regulation (as assessed by the Stroop and emotional Stroop task).

Although our study provided some insights into the association between FA, emotion regulation and obesity, it has some methodological limitations. Firstly, our samples sizes were small to moderate, which is associated with a higher probability of type II error. Secondly, we did not systematically evaluate the presence of BED, which could be a confusion factor on emotional regulation aspect of our sample. Thirdly, our control population presented some differences with our clinical sample, namely, different methods to assess height and weight (extracted from medical records for patients and self-reported for control) which could induce differential misclassification [63] and significant socio-demographic differences with our clinical population. This could have led to a confusion bias which may have been explained by our recruitment method for the control group (i.e., university members and students), with the recruitment of younger persons with higher employment rate and higher level of education. Of note, there was, however, no correlation between age and cognitive emotion regulation strategy. Fourthly, the cross-sectional nature of our study precludes us from demonstrating a causal relationship between emotional regulation strategy and FA. To demonstrate whether these cognitive emotion regulation strategies happen before, during or after the onset of FA, prospective studies are warranted. As a whole, our work open some interesting prospects on emotion regulation aspects of bariatric seeking patients with obesity and food addiction and it’s similarity with some aspect of other disorder (binge eating disorder and gambling disorder) but needs to be replicated with a more robust methodology before being considered as a specificity of food addiction.

Despite these limitations, our study has some clinical implications. Firstly, it demonstrates the need to explore emotion regulation mechanism in patients with FA through different and complementary methods such as psychometry for cognitive emotion regulation (CERQ), difficulties in emotion regulation (DERS), alexithymia (TAS-20) and neuropsychological measures. Secondly, it brings support for the use of the CERQ, a self-administered tool rarely used in the field of addictive and eating disorder,. Finally, It brings insight into emotion regulation aspects that may be linked to obesity itself and emotion regulation aspects that may be associated more specifically to FA which may represent specific targets (i.e., some specific cognitive emotion regulation strategies). In sum, our results are compatible with the hypothesis of a specific cognitive avoidance toward the exposition to negative emotions and its associated events in patients with obesity and FA. Therapeutic implications that may be helpful in these patients may include the use of psychotherapy to increase the patient’s perceived self-efficacy toward the exposition or anticipation of exposition to these emotionally charged events (i.e., Cognitive behavioral therapy including Aceptment & Commitment Therapy and Dialectical behavioral therapy) including both emotion regulation ability and cognitive strategy restructuration as already demonstrated in patients with substance use disorder [64].

## Supplementary Information

Below is the link to the electronic supplementary material.

Supplementary Material 1.

## Data Availability

The datasets used and/or analysed during the current study are available from the corresponding author on reasonable request.
