# Supplementary material for: Emotion regulation among bariatric seeking patients with obesity and food addiction: a cross-sectional, unmatched nested case–control comparison
Source: J Eat Disord. 2025 Dec 15;13:283. doi: 10.1186/s40337-025-01424-6 (PMC12706897; doi:10.1186/s40337-025-01424-6)
Supplement: Supplementary file 1 — Supplementary Material 1. [file 40337_2025_1424_MOESM1_ESM.docx]

# Supplemental material

Supplemental Material A. ::  specifications of creation regarding the neuropsychological task administered »)

The neuropsychological task was administered on a computer platform using the Psychopy software. Participants completed the task on an ASUS Zenbook 14 laptop, equipped with a 14-inch screen with a resolution of 1920*1080p and a refresh rate of 60 Hz. The stimuli were presented in uppercase in the center of a uniform gray background in the Open Sans font. The Stroop test was designed in blocks to enhance sensitivity [53].Each word was preceded by a central fixation cross presented for 500 milliseconds (ms). The font size was 0.1, corresponding to a height of 10% of the screen height. Participants were instructed to identify as quickly and accurately as possible the color of the word displayed on the screen. Response time (in ms) and response accuracy were collected.

This task was composed of 6 blocks, presented in succession. The beginning of a new block was preceded by a screen displaying the instructions for a minimum of 30 seconds, to mitigate the influence of the previous block on the emotional Stroop effect [54].Classic Stroop was composed of 3 blocks; Word Block, Color Block and Color-Word Block. Emotional Stroop was composed of Neutral Block, Emotional Block and Food Block. Each block consisted of 20 words for the emotional Stroop and 16 words for the classic Stroop. Presentation order was pseudo-randomized as illustrated in Figure X. The "Word" block was always presented first, followed by the "Color" block. The next two blocks were randomized between the "Neutral" block and the "Word-Color" block. The next two blocks were randomized between the "Food" block and the "Emotional" block.

Figure 2 Emotional Stroop Design
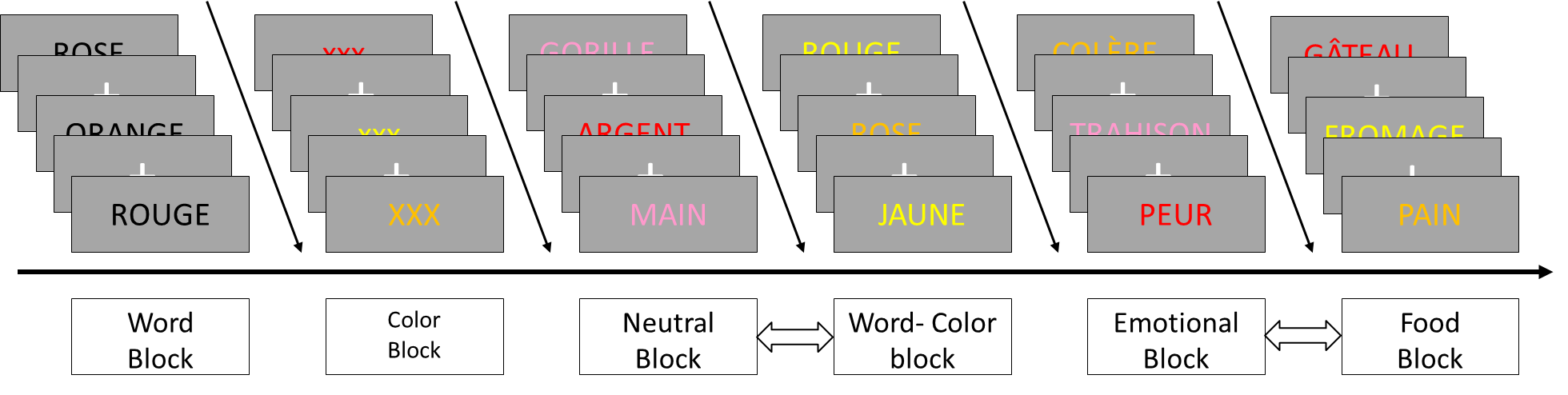


Translation of the illustrated words: ROSE: Pink; ORANGE: Orange; ROUGE : Red; GORILLE: Gorilla; ARGENT : Money; MAIN : Hand; COLÈRE : Anger; TRAHISON : Treason; PEUR: Fear; GÂTEAU: Cake; FROMAGE : Cheese; PAIN : Bread

The words used in Food Block were foods reported as subjects to loss of control in YFAS for more than 30% of patients in precedents study [23,55]. The words used in the neutral, emotional and food conditions were paired in number of letters and frequency (books and films) according to French affective lexicons [56,57] using a Kruskall-Wallis test.
